# Supplementary material for: COVID-19 Vaccine: A Potential Risk Factor for Accelerating the Onset of Bullous Pemphigoid
Source: Vaccines (Basel). 2024 Sep 5;12(9):1016. doi: 10.3390/vaccines12091016 (PMC11436231; doi:10.3390/vaccines12091016)
Supplement: Supplementary file 1 [file vaccines-12-01016-s001.zip › vaccines-3152258-supplementary.pdf]

Supplementary Materials

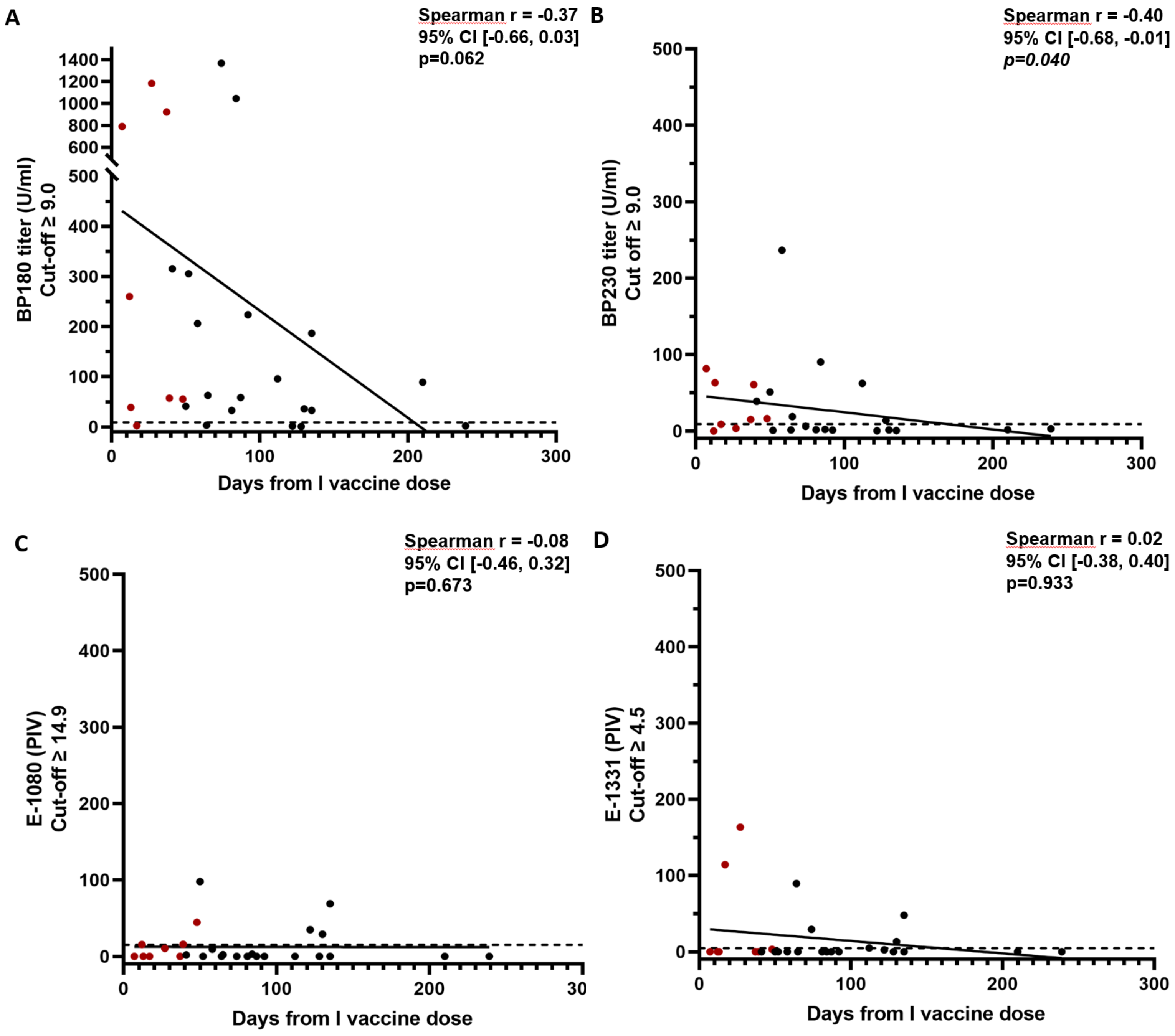

Supplementary Figure S1. Correlation of latency from first vaccine dose to BP onset and autoantibody titers. Plot of time since vaccination and IgG autoantibody titers towards (A) BP180, (B) BP230, (C) E-1080, and (D) E-1331 of 27 patients enrolled at disease diagnosis who developed bullous pemphigoid after the I or II dose of vaccine. The dotted lines represent the cut-off for each assay. The red dots represent vaccine-associated patients.

Panel A

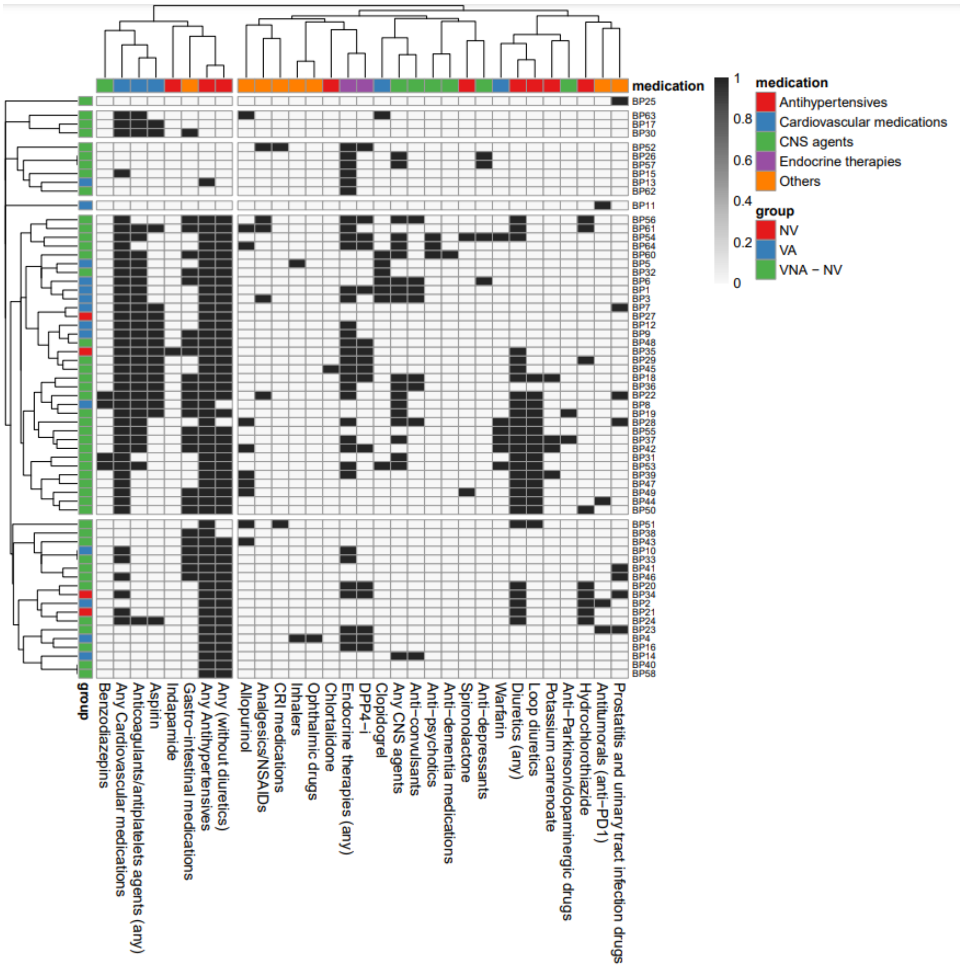

Panel B

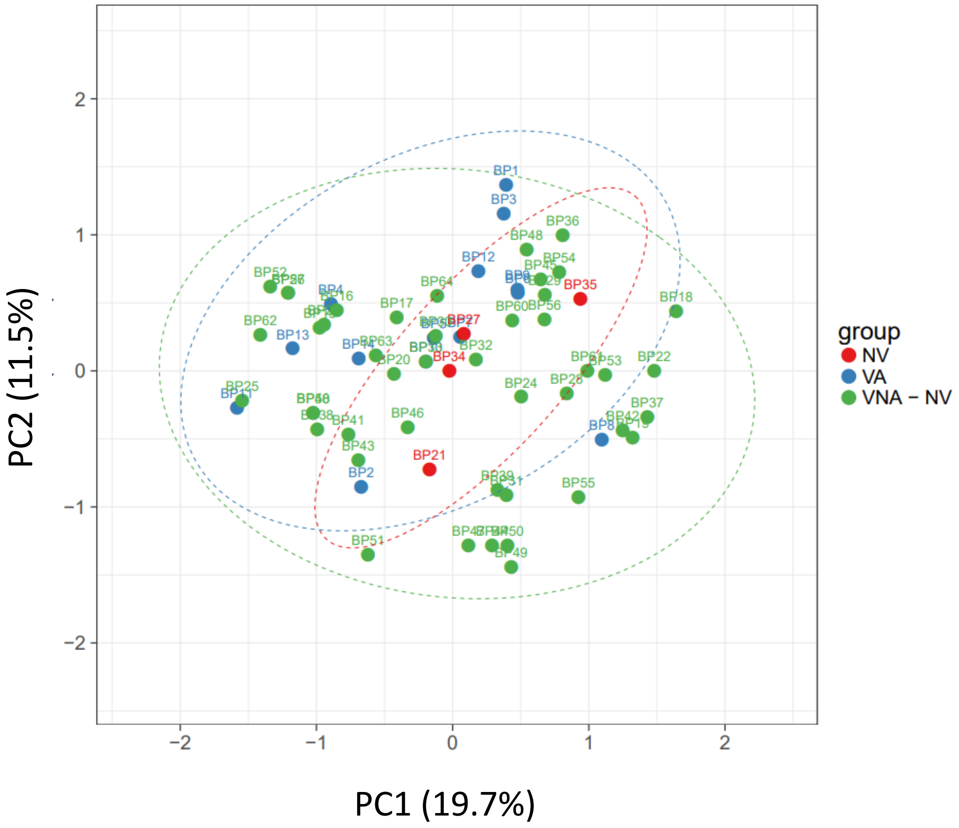

Supplementary Figure S2. Patterns and relationships within the pharmacological dataset of 64 BP patients. (Panel A), PCA of pharmacological treatment (binary) for three groups of patients ("NV": non-vaccinated; "VNA": vaccine-associated; and "VNA-NV": vaccine-non-associated, minus the non-vaccinated individuals), where the principal component explaining up to 19% of the overall variance. No scaling is applied to rows; SVD with imputation is used to calculate principal components. X and Y axis show principal component 1 and principal component 2 that explain 19.7% and 11.5% of the total variance, respectively. Dataset frame was not row-centered, while constant columns were removed. As a result, patient BP59 from the NV group was excluded from the analysis because he did not receive any of the five pharmacological treatments. (Panel B), a heatmap is presented, revealing a cluster of more commonly used drugs and three subgroups of patients (from top to bottom) based on the combinations of individual drugs used. No scaling is applied to columns. Rows are clustered using binary distance and complete linkage. Columns are clustered using correlation distance and complete linkage. 63 rows, 31 columns.

Supplementary Table S1. Short list of autoimmune diseases that have been documented following COVID-19 vaccines administration.

**Autoimmune diseases**	**COVID-19 vaccine type**	**Reference**
Alopecia areata	mRNA, viral vector, inactivated	[11]
Psoriasis	mRNA	[12]
Autoimmune bullous diseases	mRNA, viral vector, inactivated	[13]
Autoimmune thyroid disease	mRNA, viral vector, inactivated	[14]
Autoimmune myocarditis	mRNA	[15]
Immune thrombocytopenia	mRNA	[16]
Guillain-Barré syndrome	mRNA, viral vector	[17]
Rheumatoid arthritis	mRNA	[18]
Systemic lupus erythematosus	mRNA	[19]
Antiphospholipid syndrome	mRNA	[20]
Adult-onset Still’s disease	mRNA	[21]
Behçet’s disease	mRNA	[22]
Autoimmune hepatitis	mRNA	[23]
Type 1 diabetes mellitus	mRNA	[24]
Autoimmune hemolytic anemia	mRNA, viral vector	[25]

Supplementary Table S2. Clinical and immunological features of vaccine associated, vaccinated, and non-vaccinated patients enrolled at diagnosis.

	**8 VA Patients**	**37 Vaccinated Patients**	**5 Non-Vaccinated Patients**	**P value (VA vs Non-Vaccinated Patients)**	**P value (Vaccinated vs Non-Vaccinated Patients)**
**BPDAI score (mean)**	41.1	38.1	37.1	0.943	0.976
**BP180 positivity (%)**	87.5	78.4	100.0	>0.999	0.564
**BP180 titer (median U/ml)**	259.9	88.1	93.6	0.530	>0.999
**BP230 positivity (%)**	62.5	48.7	60.0	>0.999	>0.999
**BP230 titer (median U/ml)**	60.5	55.7	26.4	0.786	>0.999
**E-1080 positivity (%)**	37.5	40.5	60.0	0.592	0.636
**E-1080 titer (median PIV)**	15.7	44.6	136.5	0.100	0.302
**E-1331 positivity (%)**	25.0	32.4	80.0	0.103	0.061
**E-1331 titer (median PIV)**	138.8	48.7	40.6	0.133	0.770

BPDAI: bullous pemphigoid disease area index; VA: vaccine-associated. The 37 vaccinated patients include all the vaccinated patients of the study population enrolled at diagnosis, without distinguishing between VA or VNA (vaccine-non-associated) patients. The 5 non-vaccinated patients (all enrolled at diagnosis) are part of the VNA group. "BP180" only refers to the immunodominant region of BP180 (NC16A). P-values for BPDAI scores and autoantibody titers were calculated with the Mann-Whitney U test. P-values for reactivity to different epitopes were calculated using the Fisher's exact (probability) test.

Supplementary Table S3. Percentage of vaccine-associated and non-associated patients affected by different comorbidities.

Comorbidities	50 VNA patients	14 VA patients	P value
Hypertension	78.0%	92.9%	0.274
Neurological diseases	24.0%	28.6%	0.735
Tumors	22.0%	28.6%	0.723
Dysthyroidism	20.0%	21.4%	>0.999
Celiac disease	2.0%	0,0%	>0.999
Multiple sclerosis	2.0%	0,0%	>0.999
Cardiovascular diseases	58.0%	50.0%	0.187
Cerebrovascular accidents	16.3%	21.4%	0.696
Dyslipidemia	32.0%	28,6%	>0.999
Chronic kidney failure	16.0%	7.1%	0.670
Autoimmune diseases	4.0%	7.1%	0.529
Diabetes	50.0%	50.0%	>0.999
Diabetes complications (any)	24.0%	14.3%	0.715
Respiratory diseases (any)	10.0%	14.3%	0.641
Asthma, bronchitis, pulmonary emphysema	8.0%	14.3%	0.604
Chronic obstructive pulmonary disease	2.0%	0,0%	>0.999

VA: vaccine-associated; VNA: vaccine-non-associated. P-values were calculated using the Fisher's exact (probability) test.

Supplementary Table S4. Assessment of the relationship between four continuous autoAb titers and 16 dichotomous comorbidities using binary logistic regression. The analysis compared two groups, VNA and VA patients, to determine whether the levels of specific autoantibodies could predict the presence of various comorbidities.

Comparison	Estimate	p-value Estimate	Group Comparison Significance
BP180 over Hypertension	-0.001230528	0.2585651	Not significant
BP180 over Neurological diseases	0.000707918	0.4754494	Not significant
BP180 over Tumors	-0.002151926	0.25675	Not significant
BP180 over Dysthyroidism	0.001367819	0.2923529	Not significant
BP180 over Celiac disease	-2.71E-17	1	Not significant
BP180 over Multiple sclerosis	0.000210082	0.9478684	Not significant
BP180 over Cardiovascular diseases	-0.000569031	0.5565074	Not significant
BP180 over Cerebrovascular accidents	-0.007362111	0.2098857	Not significant
BP180 over Dyslipidemia	-0.002278589	0.2021109	Not significant
BP180 over Chronic kidney failure	0.001845736	0.1897148	Not significant
BP180 over Autoimmune diseases	-207.6885	0.8929531	Not significant
BP180 over Diabetes	0.002796345	0.07312917	Not significant
BP180 over Diabetes complications (any)	2.23E-05	0.9851651	Not significant
BP180 over Respiratory diseases (any)	0.001026864	0.4266669	Not significant
BP180 over Asthma, bronchitis, pulmonary emphysema	0.001213594	0.3832017	Not significant
BP180 over Chronic obstructive pulmonary disease	4.37E-06	0.9990006	Not significant
BP230 over Hypertension	0.006398025	0.4532296	Not significant
BP230 over Neurological diseases	0.01209629	0.06376347	Not significant
BP230 over Tumors	-0.01733822	0.2262342	Not significant
BP230 over Dysthyroidism	-0.1060262	0.2516165	Not significant
BP230 over Celiac disease	-9.89E-17	1	Not significant
BP230 over Multiple sclerosis	-1.676159	0.4486007	Not significant
BP230 over Cardiovascular diseases	0.006290763	0.3290565	Not significant
BP230 over Cerebrovascular accidents	0.004504825	0.4780627	Not significant
BP230 over Dyslipidemia	-0.002389882	0.6881717	Not significant
BP230 over Chronic kidney failure	0.01330787	0.07000554	Not significant
BP230 over Autoimmune diseases	-0.009732049	0.7612616	Not significant
BP230 over Diabetes	0.004079241	0.5047477	Not significant
BP230 over Diabetes complications (any)	-0.000765626	0.9113441	Not significant
BP230 over Respiratory diseases (any)	0.01146903	0.1068254	Not significant
BP230 over Asthma, bronchitis, pulmonary emphysema	-0.000851687	0.9441706	Not significant

BP230 over Chronic obstructive pulmonary disease	0.04127626	0.3725857	Not significant
PIV1080 over Hypertension	0.000986158	0.8319191	Not significant
PIV1080 over Neurological diseases	0.001246913	0.7796178	Not significant
PIV1080 over Tumors	-0.005086317	0.4048639	Not significant
PIV1080 over Dysthyroidism	-0.002519316	0.6972338	Not significant
PIV1080 over Celiac disease	9.14E-19	1	Not significant
PIV1080 over Multiple sclerosis	0.001824162	0.8613251	Not significant
PIV1080 over Cardiovascular diseases	-0.000463113	0.9066038	Not significant
PIV1080 over Cerebrovascular accidents	-0.01114413	0.302662	Not significant
PIV1080 over Dyslipidemia	0.004351816	0.2939738	Not significant
PIV1080 over Chronic kidney failure	-0.09186795	0.4624505	Not significant
PIV1080 over Autoimmune diseases	-12.84991	0.9956031	Not significant
PIV1080 over Diabetes	3.31E-05	0.9933446	Not significant
PIV1080 over Diabetes complications (any)	-0.000888936	0.8539888	Not significant
PIV1080 over Respiratory diseases (any)	-0.003845222	0.6699137	Not significant
PIV1080 over Asthma, bronchitis, pulmonary emphysema	-0.000729809	0.9336227	Not significant
PIV1080 over Chronic obstructive pulmonary disease	-0.09802095	0.6871813	Not significant
PIV1331 over Hypertension	-0.003494633	0.7008804	Not significant
PIV1331 over Neurological diseases	-0.00222678	0.7984017	Not significant
PIV1331 over Tumors	-0.0160988	0.2535511	Not significant
PIV1331 over Dysthyroidism	-0.02630616	0.3659549	Not significant
PIV1331 over Celiac disease	2.03E-16	1	Not significant
PIV1331 over Multiple sclerosis	0.01715789	0.4446176	Not significant
PIV1331 over Cardiovascular diseases	-0.008987232	0.2694224	Not significant
PIV1331 over Cerebrovascular accidents	-0.02117983	0.2619115	Not significant
PIV1331 over Dyslipidemia	0.01736833	0.06683686	Not significant
PIV1331 over Chronic kidney failure	0.006246135	0.6917422	Not significant
PIV1331 over Autoimmune diseases	-20.26923	0.9961276	Not significant
PIV1331 over Diabetes	0.007872359	0.3379121	Not significant
PIV1331 over Diabetes complications (any)	-0.0123803	0.3682906	Not significant
PIV1331 over Respiratory diseases (any)	0.009202476	0.3906748	Not significant
PIV1331 over Asthma, bronchitis, pulmonary emphysema	0.01282587	0.2642214	Not significant
PIV1331 over Chronic obstructive pulmonary disease	-0.03966363	0.6875065	Not significant

autoAb: autoantibodies; VA: vaccine-associated; VNA: vaccine-non-associated. P-values were calculated using binary logistic regression analyses.
